# Effective treatment using itraconazole combined with terbinafine in the treatment of nasal sporotrichosis: A case report

**DOI:** 10.1097/MD.0000000000017155

**Published:** 2019-09-13

**Authors:** Keying Guo, Shenghua Wang, Zhenying Wang, Li Zhang

**Affiliations:** a Department of Dermatology; b Department of Clinical Laboratory, Shandong Provincial Hospital Affiliated to Shandong University, China.

**Keywords:** itraconazole, sporotrichosis, terbinafine

## Abstract

**Rationale:**

Sporotrichosis is a subacute or chronic infection caused by sporothrix schenckii complex. The misdiagnosis rate of sporotrichosis is very high. Fungal microscopic examination and timely culture help us make an accurate diagnosis and treatment. We observed that combined treatments are more effective than monotherapy in treatment of sporotrichosis.

**Patient concerns:**

A 47-year-old female complained of pustules and scabs on her nose tip that lasted for 1 month at our hospital. She was diagnosed with skin infection and treated with antibiotics for 20 days. Nonetheless, the treatment did not result in any improvement with the lesion.

**Diagnoses:**

The results on bacterial culture, sensitive test, special stains, and multiple acid-fast cultures were negative. Finally, fungi were observed by KOH. Finally, fungal hyphae were observed by KOH and by fluorescent staining. Taupe filamentous colonies of sporothrix-like species appeared by fungal culture. The diagnosis of sporotrichosis was finally confirmed based on the lesion characteristics and the results of laboratory examination.

**Interventions:**

The lesions did not alleviate with Itraconazole oral administration for 1 month. Then we treated the patient with the combination therapy of itraconazole (ITR) and terbinafine. At the same time, the compound glycyrrhizin tablet was used for liver protection.

**Outcomes:**

The patient was free of clinical symptoms of sporotrichosis following the treatment and did not have complications during an 8-month follow-up.

**Lessons:**

We should always be alert to sporotrichosis although it is not a very common disease. It is important to adapt fungi microscopic analysis and culture for an accurate diagnosis. ITR is the first choice for sporotrichosis. However, combination treatment is more effective for stubborn cases.

## 1 Introduction

Sporotrichosis, a subacute or chronic infectious disease caused by infection with sporothrix schenckii complex, consists of cutaneous sporotrichosis (fixed cutaneous, lymphocutaneous, and multifocal or disseminated cutaneous sporotrichosis) and outer cutaneous sporotrichosis.^[1,2]^ The condition even can spread the whole body, causing multiple system damage. Sporothrix schenckii complex exists extensively on the soil, surfaces of wood and plant, and invade the body through skin lesions, such as scratches or bite wounds of plant stem punctures or fungal-carrying animals (mainly felines). Even visceral organs can be involved.^[3]^ There are many drugs for the treatment of sporotrichosis, itraconazole (ITR), amphotericin B, terbinafine (TB), potassium iodine, and so on. ITR is currently the first choice for the treatment of cutaneous Sporidiosis. Olga C. Rojas’ research showed that TB had the best antifungal activity in vitro in the treatment of sporofiliosis.^[4]^ This report presents a case of Nasal sporotrichosis in the treatment which ITR combined with TB are more effective than ITR alone. At the same time, basic medical institutions should enhance awareness of fungal infections so as not to delay the best treatment period.

## 2 Case report

**Figure 1:**
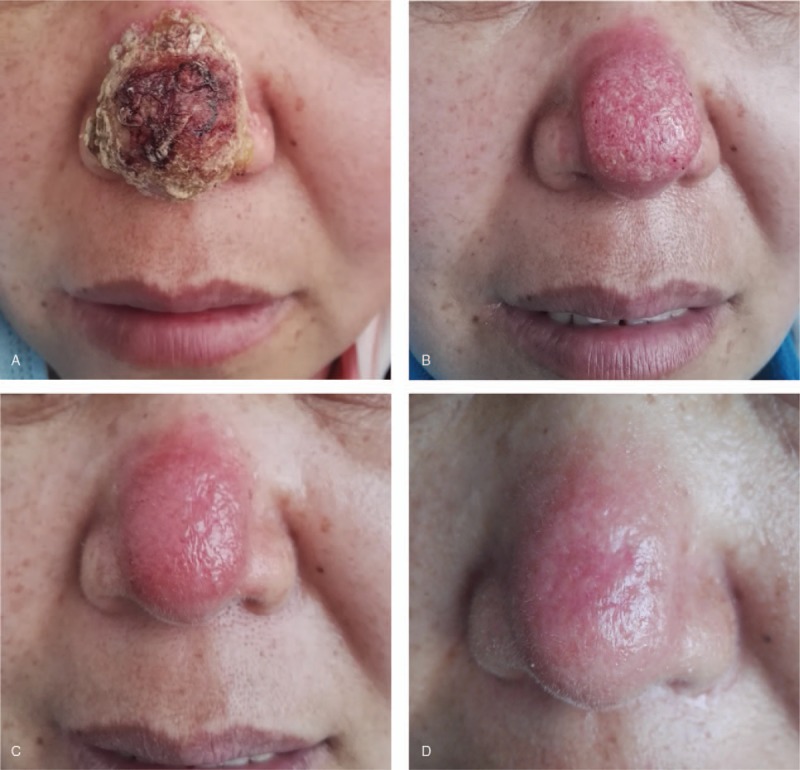
Manifested a plaque on the tip of the nose about 2 cm × 3 cm, which surface was covered with tawny scabs (A). Treatment by ITR for 1 month (B). Then ITR combined with TB for 1 month (C). ITR combined with TB for 3 months, clinical cure was obtained (D). ITR = Itraconazole, TB = Terbinafine.

**Figure 2:**
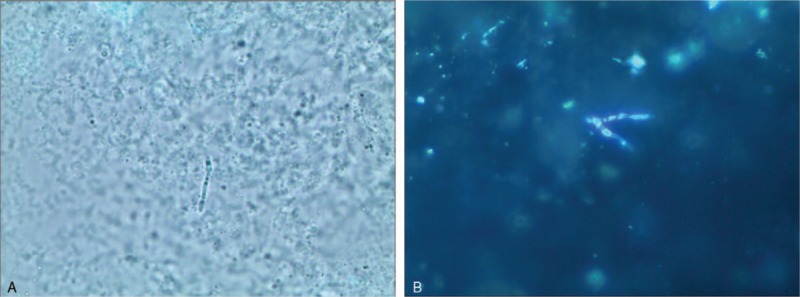
Fungal hyphae was observed by KOH (Potassium hydroxide) (×40) (A) and by fluorescent staining (×40) (B).

**Figure 3:**
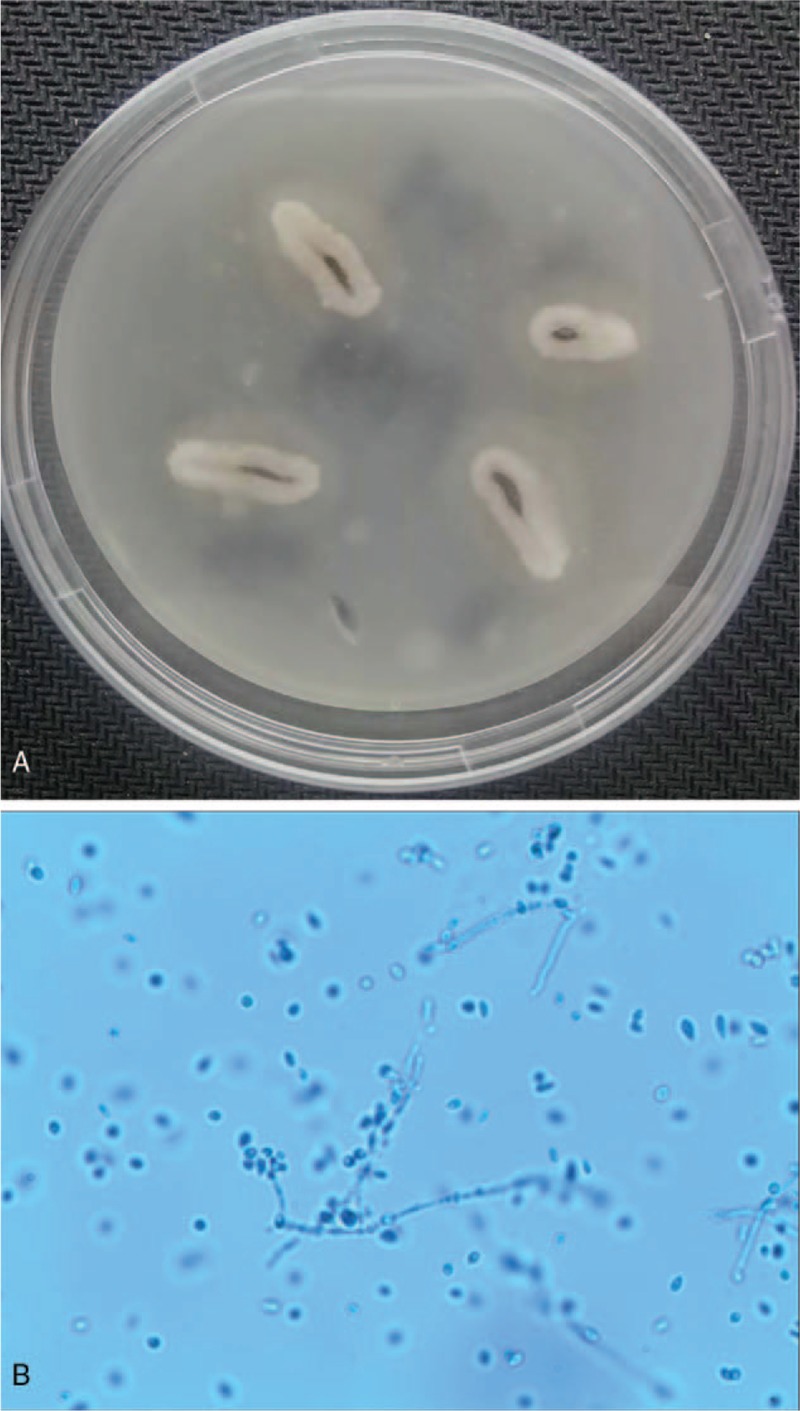
Fungal culture for 2w, grew taupe filamentous colonies of Sporothrix-like species (A). The sporothrix was clustered (×400) (B).

A 47-year-old female presented pustules and scabs on her nose tip for 1 month, with itching and pain. An isolated “small blister” appeared at the tip of the patient's nose 1 month ago before the visit. After the scratch, the blisters increased, and papules appeared, gradually forming a plaque with pustules and scabs, which forming ulcers (Fig. 1A). Basic medical institutions were treated with “penicillin” for 20 days for anti-inflammation (other drugs and doses were unknown), while ineffectively. Instead, the skin lesions progressively thickened, expanded and eroded, irregularly accompanied with itching and pain. In order to clearer diagnosis and better treatment, the patient came to outpatient in the dermatology department. She had no other history except hepatitis B. The dermatological examination revealed that there was an ulcerated plaque on the nasal tip which was 2 cm × 3 cm and covered with crusta. The ulcer surface presented cobblestone vegetans and was surrounded by a rim of erythema and pustules. (Fig. 1A). There were no papules and lumps in the face and neck, and no enlarged lymph nodes in the submandibular and neck. Due to clinical suspicion, our diagnosis is a skin bacterial or fungal infection, treatment with ITR 200 m/d was initiated. The results on bacterial culture and sensitivity test of pustule contents and tissue exudates were negative. Similarly, special stains also failed to reveal any acid-fast bacilli, and multiple acid-fast tissue cultures were negative. However, samples from effusion of pustules were screened by KOH microscopic and fluorescent staining (FS) examination. We observed fungi by KOH (Fig. 2A) and fluorescently labeled hyphae by FS (Fig. 2B). We harvested the pyogenic fluids at 37°C for fungal culture, after 2w, which grew taupe filamentous colonies of sporothrix-like species (Fig. 3A). Through the microscope, we observed that the sporothrix was clustered (Fig. 3B). According to auxiliary examination, we confirmed the previous diagnosis (sporotrichosis, fixed cutaneous) and increased the ITR dosing regimen to 300 m/d, 2w. The patient did not show signs of clinical improvement (Fig. 1B).

In order to shorten the time of therapy, we considered combining ITR with TB (250 m/d) while the lesion was removed for 1 month (Fig. 1C). As the patient's HBsAg, HBeAb HBcAb tests were positive, closely monitored liver function while we reduced the ITR dosing regimen to 200 m/d, two months, and the clinical cure was obtained (Fig. 1D). During the treatment, the patient had been taking Compound Liquorice Tablets to protect liver function. And the patient was free of clinical symptoms of sporotrichosis following the treatment and did not have complications during 8-month follow-up.

## 3 Discussion

Lymphocutaneous sporotrichosis is the most common clinical form globally. But in China, the fixed cutaneous form is prevalent, followed by the lymphocutaneous form.^[5]^ It is high degrees of endemicity for sporothrix species, although sporotrichosis is globally distributed.^[6]^ According to the data in existing literature, Brazil, China, and South Africa are the most endemic regions, and incidences of sporotrichosis in rural areas are higher than that in the city.^[7]^

This case needs to be differentiated from some diseases, such as bacterial infected skin diseases, other fungal skin diseases, and non-infectious skin diseases.^[8]^ Fungal culture examination is the gold standard for the diagnostic confirmation of sporotrichosis.^[9]^ As regards the reason for misdiagnosis of this patient may be related to the following factors:

(1) This patient had no history of traumatic injury or foreign body implantation, and the skin lesion was in the nose,^[8]^ often leading to the ignorance of the infection of deep mycosis;
(2) Basic medical institutions ignored the regional distribution characteristics of sporotrichosis in urban and rural areas.^[10]^

Many countries strictly follow the dosing regimen of ITR 200 m/d for 3 to 6 months to treat cutaneous sporotrichosis. If the whole treatment was calculated as a percentage, the patient's symptom was relieved by 5% in the first month, but it was surprising that the skin lesion had been relieved by 30% one month after drug combination. For species of Sporothrix, some clinical isolates are considered possibly resistant to ITR,^[11]^ therefore, when the effect of ITR alone on the treatment of cutaneous sporomycosis is poor, it is suggested that ITR and TB should be used together. This is in agreement with the results of Olga C. Rojas’ research.^[4]^

Considering that drugs may impair liver function, we strictly test liver function and protect it with drugs. During the entire treatment period, the patient's liver enzymes have never been abnormal. This result suggests that liver function drugs are protective, and it is also suggested that ITR and TB are safe for the treatment of patients with chronic hepatitis B.

To explore the curative effect of combination therapy with ITR and TB on cutaneous sporotrichosis is intriguing, and the dosage standards for both drugs also need further study. This case report is not a controlled clinical trial, so more cases and practice support are needed.

## Author contributions

**Conceptualization:** Zhenying Wang.

**Data curation:** Keying Guo, Shenghua Wang, Li Zhang.

**Investigation:** Shenghua Wang, Li Zhang.

**Visualization:** Zhenying Wang.

**Writing – original draft:** Keying Guo.

**Writing – review & editing:** Keying Guo, Zhenying Wang.

## References

[1] Mahmoudi S Zaini F Kordbacheh P Sporothrix schenckii complex in Iran: Molecular identification and antifungal susceptibility. Med Mycol 2016;54:593–9. 26933207 10.1093/mmy/myw006

[2] Vettorato R Heidrich D Fraga F Sporotrichosis by Sporothrix schenckii senso stricto with itraconazole resistance and terbinafine sensitivity observed in vitro and in vivo: case report. Med Mycol Case Rep 2018;19:18–20. 29204336 10.1016/j.mmcr.2017.10.001 PMC5711665

[3] Lopesbezerra LM Moramontes HM Zhang Y Sporotrichosis between 1898 and 2017: the evolution of knowledge on a changeable disease and on emerging etiological agents. Med Mycol 2018;56:126–43. 29538731 10.1093/mmy/myx103

[4] Rojas OC Bonifaz A Campos C Molecular Identification, antifungal Susceptibility, and geographic origin of clinical strains of Sporothrix schenckii Complex in Mexico. J Fungi 2018;4:86. 10.3390/jof4030086 PMC6162654 30036959

[5] Chen M Xu Y Hong N Epidemiology of fungal infections in China. Front Med 2018;12:58–75. 29380297 10.1007/s11684-017-0601-0

[6] Zhang Y Hagen F Wan Z Two cases of sporotrichosis of the right upper extremity in right-handed patients with diabetes mellitus. Rev Iberoam Micol 2016;33:38–42. 25982353 10.1016/j.riam.2015.02.001

[7] Zhang Y Hagen F Stielow B Phylogeography and evolutionary patterns in Sporothrix spanning more than 14 000 human and animal case reports. Persoonia 2015;35:1–20. 26823625 10.3767/003158515X687416 PMC4713101

[8] Orofino-Costa R Macedo PM Rodrigues AM Sporotrichosis: an update on epidemiology, etiopathogenesis, laboratory and clinical therapeutics. An Bras Dermatol 2017;92:606–20. 29166494 10.1590/abd1806-4841.2017279 PMC5674690

[9] Barros MB de Almeida Paes R Schubach AO Sporothrix schenckii and Sporotrichosis. Clin Microbiol Rev 2011;24:633–54. 21976602 10.1128/CMR.00007-11 PMC3194828

[10] Sanchotene KO Madrid IM Klafke GB Sporothrix brasiliensis outbreaks and the rapid emergence of feline sporotrichosis. Mycoses 2015;58:652–8. 26404561 10.1111/myc.12414

[11] Espinelingroff A Abreu DPB Almeidapaes R Multicenter, international study of mic/mec distributions for definition of epidemiological cutoff values for sporothrix species identified by molecular methods. Antimicrob Agents Chemother 2017;61: AAC.01057-17. 10.1128/AAC.01057-17 PMC5610517 28739796

